# Quantifying the adhesive strength between the SARS-CoV-2 S-proteins and human receptor and its effect in therapeutics

**DOI:** 10.1038/s41598-020-74189-4

**Published:** 2020-10-16

**Authors:** Mauricio Ponga

**Affiliations:** Department of Mechanical Engineering, University of British Columbia, 2054-6250 Applied Science Lane, Vancouver, BC V6T 1Z4 Canada

**Keywords:** Endocytosis, Cell adhesion, SARS-CoV-2, Mechanical engineering

## Abstract

The binding affinity and adhesive strength between the spike (S) glycoproteins of the severe acute respiratory syndrome coronavirus 2 (SARS-CoV-2), and the human angiotensin-converting enzyme 2 (ACE2) receptor is computed using molecular dynamics (MD) simulations. The calculations indicate that the binding affinity is $e_{RS}= 12.6 \pm 1$ $\hbox {kCal}{\cdot }\hbox {mol}^{-1}$ with a maximum adhesive force of $\sim 102$ pN. Our analysis suggests that only 27 (13 in S-protein, 14 in ACE2) residues are active during the initial fusion process between the S-protein and ACE2 receptor. With these insights, we investigated the effect of possible therapeutics in the size and wrapping time of virus particles by reducing the binding energy. Our analysis indicates that this energy has to be reduced significantly, around 50% or more, to block SARS-CoV-2 particles with radius in the order of $R\le 60$ nm. Our study provides concise target residues and target binding energy reduction between S-proteins and receptors for the development of new therapeutics treatments for COVID-19 guided by computational design.

## Introduction

In late 2019 a novel severe acute respiratory syndrome coronavirus (named SARS-CoV-2) was infecting people in China, causing severe pneumonia^1^. Within a few months from the first outbreak, the novel coronavirus created a global pandemic that forced the majority of the world’s population under lockdown. The novel SARS-CoV-2 virus keeps infecting and killing a large number of people around the globe. Thus, it is imperative to understand and develop therapies that can combat COVID19, the illness caused by the SARS-CoV-2 coronavirus.

SARS-CoV-2 belongs to the $\beta -$coronavirus genus^2^ and they usually enter to host cells by attaching and fusing to the cell membrane^3^. Cell receptors diffuse across the membrane’s external surface to reach proximity with the virus’ proteins, binding together for their posterior fusion, promoting membrane bending and virus wrapping until final uptake. Coronaviruses affinity with cell receptors occurs via a transmembrane spike (S) glycoprotein forming homotrimers on the virus’ capsid^2,4^. The S-protein is made of two functional subunits (S1 and S2) responsible for fusion to the viral-receptor adhesion. Due to their critical role in SARS-CoV-2 infections, S-proteins are the common target for developing antibodies and therapeutics for COVID19.

Several works have thus far been focused on characterizing the S-protein and its trimeric structure using cryoEM techniques. For instance, Wrapp et al.^5^ have provided a cryoEM structure in the prefusion conformation and have found two states, labeled as *up* and *down*, whereby the S1 subunit is exposed and retracted, respectively. The exposed region that links to the human receptor is known as the receptor-binding domain (RBD). At the same time, Lan et al.^4^ have studied the RBD bounded to the ACE2 and provided a detailed description of the S1 subunit that compose the RBD and its link to the ACE2 receptor. In particular, they found that the S-protein links to the N-terminus helix of the ACE2 protein serving as an anchor point. Moreover, only a reduced number of residues, in total 20, were in close contact with the ACE2 terminus helix and even a smaller portion was within 0.4 nm from it^4^. This observation suggests that the adhesive forces arise through short-range interactions (e.g., Hydrogen bonds and salt bridges) between these two proteins. Shang et al.^6^ provided a structural basis for receptor recognition of SARS-CoV-2. They found that in addition to the residues in the S1 subunit, many glycans generated links between the two proteins. Understanding the link between the S-protein and ACE2, and in particular, the RBD is key to tackling the pandemic caused by SARS-CoV-2.

The importance of the S-protein/ACE2 interface has motivated researchers to explore the phenomenon with both experimental and computational methodologies due to a growing interest in repurposing therapeutic to treat COVID19. However, testing the efficacy of these drugs is time consuming and expensive, pushing scientists to develop predictive models based on computational tools to reduce development time. For instance, Smith et al.^7^ have scanned thousands of ligands with molecular dynamics simulations of the RBD, and have ranked these ligands based on their affinity. Other studies have focused their attention on quantifying the S-protein/ACE2 receptor’s formation energy, using a full trimeric model and/or a single S-protein/ACE2 receptor^8–11^. While these studies provide useful information on the compound’s formation energy, they failed in predicting realistic interaction energies that can be indirectly contrasted with experimental measurements. This shortcoming is because the adhesive interactions between the S-proteins and receptors are short-range, and it changes as a function of the separation length. Panda et al.^12^ pursued a similar approach to benchmark drugs and antibodies for SARS-CoV-2. These studies focused their attention on the binding affinity of chemical compounds to reduce the formation energy between SARS-CoV-2 S-protein/ACE2 receptor. However, a quantitative evaluation of the binding affinity between the S-proteins and ACE2 receptors, the adhesive strength of this bond and the chemo-mechanical determinants controlling coronavirus uptake are still missing. This knowledge gap significantly limits the impact of the aforementioned investigations and underlines the importance of the proposed study.

In this work, we investigate the chemo-mechanical interaction between S-protein and ACE2 receptors, and the resulting implications on the mechanisms for virus uptake. We computed the binding affinity and adhesive force between S-protein and ACE2 receptors and analyzed the residues in contact during the bond-breaking process. Surprisingly, our results indicate that the residues in contact change as the two proteins were pulled apart, elucidating target points to develop new therapeutics. With these findings, we investigated SARS-CoV-2 uptake to predicted the effects of the binding affinity perturbations on the uptake kinematics.

## Results and discussions

**Figure 1:**
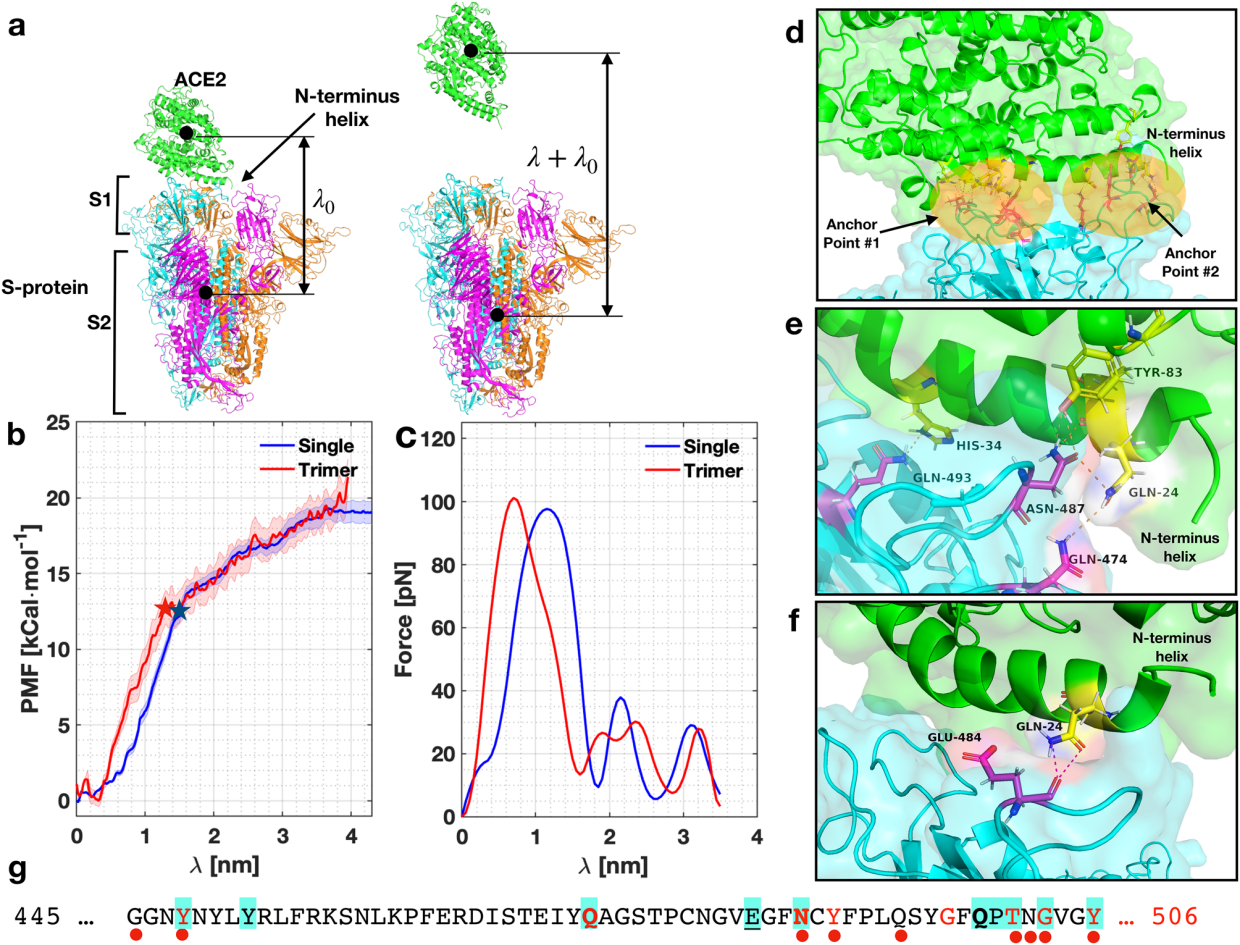
Evolution of the PMF, force, and residues with $\lambda$. (**a**) Schematic of the setup. (**b**,**c**) Evolution of the PMF and force as a function of $\lambda$ for a single S-protein/ACE2 receptor (blue) and for the trimeric protein (red). Shaded area in (**b**) represents the error band for both simulations. The stars indicate the moment when detachment has happened. (**d**) View of the two anchor points between S-protein/ACE2 receptor when $\lambda = 0.3$ nm. (**e**) Interacting residues for $\lambda = 0.8$ nm and (**f**) $\lambda = 1.4$ nm. (**g**) List of residues that are active during the pulling simulation in the RBD. Letters in red, cyan background, bold and underlined correspond to residues in contact at $\lambda = 0.3, ~0.4, ~0.8, ~1.4$ nm, respectively. Red dots indicate residues identified experimentally by Lan et al.^4^. Residue K-417 has been omitted in the sequence for shortness.

After performing umbrella sampling simulations on the S-proteins/ACE2 receptor configuration, we investigated the potential of mean force (PMF) evolution as a function of the pulling distance, i.e., the reaction coordinate ($\lambda$) (see Fig. 1a for a schematic). Figure 1b shows the results for the single and full S-protein/ACE2 receptor configuration. Focusing our attention on the full trimeric protein, we observed that, initially, the evolution of the PMF shows a metastable and a global minimum between $\lambda = 0-0.4$ nm. These configurations were separated by a small barrier of $\sim 1.5$ $\hbox {kCal}{\cdot }\hbox {mol}^{-1}$. For $0.4 \le \lambda \le 1.4$ nm, the PMF’s evolution shows almost a linear behavior with the reaction coordinate up to approximately $\lambda = 1.4$ nm, where the PMF reached $\sim 12.6 \pm 1$ $\hbox {kCal}{\cdot }\hbox {mol}^{-1}$. Thereafter, the PMF changes slopes and tends to plateau around $\sim 20 \pm 1$ $\hbox {kCal}{\cdot }\hbox {mol}^{-1}$ at the end of the sampling, when $\lambda \ge 4$ nm. The maximum error in the measure of the PMF is approximately $\sim \pm 1$ $\hbox {kCal}{\cdot }\hbox {mol}^{-1}$.

The PMF’s change at around $\lambda = \sim 1.5$ nm indicates that at this point, all van der Waals interactions are off between the two proteins, as shown with the change of slope in the plot. This was also confirmed by analyzing the residues in contact (below). Remarkably, the position where the change of slope happens is very close to the selected cutoff of the van der Waals interaction set up in our model. The remaining interactions appear due to long-range electrostatic forces that are in the model. We take the curve’s inflection point as the value of the adhesive strength that characterizes the link between the S-protein/ACE2 receptor. The result obtained with a single S-protein/ACE2 receptor leads to approximately the same free energy but a slightly different path. The binding affinity is estimated to be $e_{RS} = 12.6 \pm 1\, \hbox {kCal}{\cdot }\hbox {mol}^{-1}$ for the full trimeric model, and $e_{RS} = 12.55 \pm 0.7\, \hbox {kCal}{\cdot }\hbox {mol}^{-1}$ for the single S-protein/ACE2 receptor indicated with stars in the plot.

The force separation ${\mathbf {F}}$ between the S-protein and ACE2 receptor is obtained from the relation ${\mathbf {F}} = d e_{RS}/ d\lambda$, hence the slope of the PMF curve in Fig. 1b. The force evolution is shown in Fig.  1c for both models. We observed that the force builds up to a maximum of ${\mathbf {F}}_{max} \sim 102$ pN denoting the rupture force between the S-protein/ACE2 receptor bond, in the order of magnitude expected for adhesion in cells^13^. Thereafter, the force drops significantly due to lack of contact between the residues.

The binding energy between the S-proteins/ACE2 receptor can be used to compute the dissociation constant $$K_D = 1.32 $$ nM (see Supplementary information (SI) for calculation of the constant). Recent works have estimated the dissociation constant of the SARS-CoV-2 virus in experimental setups, obtaining values between $$1.2 \pm 0.1$$ nM to 4.674 nM^2,4^. The almost five-fold discrepancy range among previous measurements underlines the difficulty in obtaining accurate experimental data and, also, remarks the potential impact of our computational method. Considering that our model is limited to only a small portion of the real virus/cell receptor, the agreement between the simulations and experiments is remarkable and gives confidence in our computational approach.

### Molecular analysis of the adhesive mechanics

We now focus our attention on the molecular interactions between S-protein and ACE2 receptor as a function of the reaction coordinate $\lambda$. The analysis of the interactions was performed with the full model at coordinates $\lambda = \sim 0.3,~0.4,~0.8,~1.4,~1.6$ nm. Using the most typical cluster configurations (see “Methods”), we first obtained the interface residues between the two molecules and performed a contact analysis between that group. We disregarded all residues whose distance was more than 0.4 nm. As expected, the number of interactions decreased when $\lambda$ increased.

First, we analyze the configuration with minimum free energy in our simulations, corresponding to $\lambda = 0.3$ nm, shown in Fig. 1d. The S-protein anchors from two locations; namely, the ends of the N-terminus helix in the ACE2 receptor and it could reach the helix on top if it, as shown in Fig. 1d. We observed that the interacting residues in the S-protein were located between positions 417-505 of the sequence, namely residues K-417, Y-449, Q-474, N-487, Y-489, G-496, T-500, G-502, Y-505, as shown in Fig. 1g—using a one-letter sequence—with red letters (see also Figure SI1, Table SI1 and Video SI1 in the SI). These residues linked to residues Q-24, D-30, E-37, Y-41, Q-42, Y-83, K-353, G-354, D-355, R-357 in the ACE2 receptor. When $\lambda =0.4$ nm, ten residues were active—five were the same—(denoted with a cyan background in Figure 1g) with a graphical representation in Figure SI1. When $\lambda = 0.8$ nm, we observed interactions between the terminus helix and the one on top, as shown in Figure 1e involving Q-493, N-487, and Q-474 in the S-protein (bold letters in Fig. 1g), and Q-24, H-34, Y-83 in the ACE2 receptor. These residues were the most persistent ones, generating stronger links than other residues through the bond-breaking simulation. Thus, these residues can be targeted in new therapeutics strategies in COVID19. For $\lambda =1.4$ nm, we found that only two residues were interacting, namely E-484 and Q-24 in the S-protein and ACE2 receptor, respectively (underlined in Fig. 1g). Figure 1f shows the links between these residues (see SI Video SI2). For $\lambda \ge 1.6$ nm, no contacts were found.

From the 27 residues in the RBD, our simulations indicate that 13 were active during the pulling simulation. In particular, we identified 13 unique residues that were active in the S-protein, and 14 in the ACE2 (see Table SI1). These residues are the same to the ones identified by Lan et al.^4^ in their cryoEM analysis, with the exception of G-446, N-501 and Q-493. However, we did identify G-502 and Q-498, which are very close to the previously mentioned residues. This remarkable agreement gives confidence to our approach and simulations.

### Uptake modeling and the effect of therapeutics

We now analyze the effect of the binding affinity in the endocytosis of the virus in cells. We recur to the chemo-mechanical model developed by Gao et al.^14^. The model considers the bending energy of the cell membrane, the release of chemical energy during the fusion of S-protein and receptor, the configurational entropy, and the ratio between receptor and S-protein density ${\tilde{\xi }} =\xi _R/\xi _S$ (see “Methods”). Also, the model needs specific parameters that are characteristic for each virus. We discuss these parameters—which are summarized in Table 1—for SARS-CoV-2 in “Methods”.

We found that the model predicts a minimum radius of $R_{min}=27$ nm with an optimum of $R_{op} = 30$ nm at which the uptake time is minimum (around $t_w^{min} \sim 3$ s, see Figure SI2) for ${\tilde{\xi }}=0.1$. Particles below $R_{min}$ cannot be wrapped because the uptake is not energetically allowed. For smaller values of ${\tilde{\xi }}$, we found that the minimum radius increases, in particular, for a ${\tilde{\xi }} = 0.0001$ the minimum and optimal radius are $R_{min} = 34$ nm, and $R_{op}=38.5$ nm, respectively with a minimum wrapping time $t_w^{min} \sim 15000$ s, see Figure SI2. These trends are in agreement with Gao’s findings^14^. The predicted minimum radius of $\sim 30$ nm is in close agreement with experimental observations of SARS-CoV-2 particle size^1,15^ where the minimum particle radius was 30 nm. This constraint could be implicit in the molecular architecture of the virus. However, our predictions suggest that viral particles that are smaller than $\sim 30$ nm cannot be uptaken, hence preventing their reproduction inside the host cell. Moreover, the predicted optimal radius of $30-40$ nm is close to the average particle size measured experimentally of $R_{ave} = 50$ nm^1,15^.

**Table 1:** Parameters used to model the endocytosis process in an infinite membrane. The reference temperature was taken as $T=310.15$ K. *R* is the radius of SARS-CoV-2 particles, $\xi _S$ is the density of spike proteins, *D* is the diffusivity of receptors, *B* is the bending modulus of lipid bilayer, $e_{RS}$ is the binding affinity, and ${\tilde{\xi }}$ is the ration between receptor and S-protein density.

*R* [nm]	$\xi _S \,[\mu \hbox {m}^{-2}$]	$D\, [\mu \hbox {m}^{2}{\cdot }\hbox {s}^{-1}$]	$B\, [\hbox {kCal}{\cdot }\hbox {mol}^{-1}$]	$e_{RS}\, [\hbox {kCal}$]	${\tilde{\xi }} = \frac{\xi _R}{\xi _S}$
30-70	2930	0.01	12.3	12.6	$10^{-1} - 10^{-4}$

Current attempts to treat COVID19 aim to repurposing therapeutics drugs and antibodies to bind between S-proteins and ACE2 receptors, thereby reducing $$e_{RS}$$^7,12^. Next, we provide an estimation of the reduction of $$e_{RS}$$ needed to increment $$R_{min}$$ above the radius of SARS-CoV-2 particles (i.e., to stop particles from being uptaken). To this end, we modified the S-protein/receptor binding affinity $$e_{RS}^* = k e_{RS} $$, where $$k \in [0,1]$$ is a reduction factor giving no affinity for $$k=0$$ and full affinity when $$k=1$$. Thus, *k* represents the effectiveness of the treatment in reducing the binding affinity between S-proteins and ACE2 receptors.

**Figure 2:**
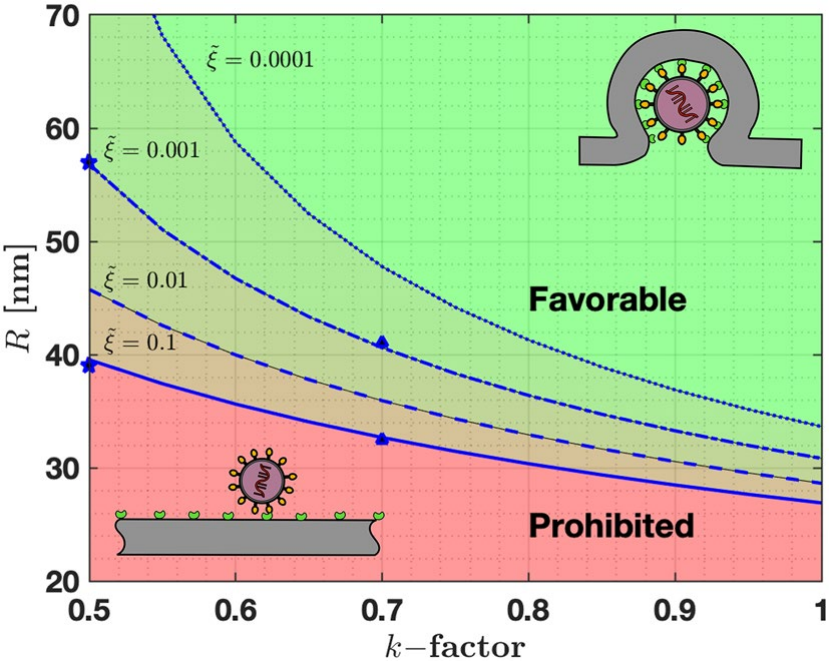
Effect of reduced binding affinity ($k-$factor) in the size of particles that can be wrapped to the cell membrane. Four receptor densities were used, namely ${\tilde{\xi }}=0.1$ (solid line), ${\tilde{\xi }}=0.01$ (dashed line), ${\tilde{\xi }}=0.001$ (dashed-dotted line), ${\tilde{\xi }}=0.0001$ (dotted line). Prohibited particles are in the red zone, while favorable particles are shown with in green. In between these zones, particle will be blocked depending on the relative receptor/S-proteins density ${\tilde{\xi }}$.

Figure 2 shows the relation between $R_{min}$ and *k* for various ${\tilde{\xi }}$. The green region identifies all particles radii for which virus uptake is always permitted independently of any reduction in binding affinity (*k*). The red region identifies particles whose radii are not permitted to be uptaken due to excessive bending energy. Between these regions, virus uptake is controlled by ${\tilde{\xi }}$. For instance, if the binding affinity between S-proteins and ACE2 receptors is reduced by 30% (k=0.7), the minimum radius of particles that can be uptaken increases by 21.5% for ${\tilde{\xi }}=0.1$ ($R_{min}^{k=0.7} =33$ nm), and by 32% for ${\tilde{\xi }}=0.001$ ($R_{min}^{k=0.7} =41$ nm, blue triangles in Figure 2). Another important aspect is that the wrapping time required for final uptake increases about 50 to 100% for these cases, respectively (see Figure SI3). This increment in the time needed for final uptake might be critical in some cases since it might give the immune system extra time to combat the infection, thus illustrating the effect of the treatment.

If we reduce the binding affinity by 50% ($k=0.5$), the minimum radius increases by 47% when ${\tilde{\xi }}=0.1$ ($R_{min}^{k=0.5} =39$ nm), and by 84% when ${\tilde{\xi }}=0.001$ ($R_{min}^{k=0.5} =57$ nm, blue stars in Fig. 2). The wrapping time also increases by 150% to 425% with respect to the case when $k=0.5$ (see Figure SI3) and allowing additional time to stop infection in cells. Finally, our work provides a rough estimation of how much binding affinity has to be reduced to provide effective treatments. Our analysis indicates that a 50% reduction in $e_{RS}$ might stop the adhesion of SARS-CoV-2 particles with $R\le 60$ nm. Moreover, we predict that smaller particles ($R \le 60$ nm) are more suitable to be blocked in comparison with larger ones, based on bending energy analysis.

## Conclusions

Our study reports the first attempt (to the author’s best knowledge) to evaluate the binding affinity and bond-breaking force between SARS-CoV-2 spike proteins and ACE2 receptors via computational analysis using an all-atom MD model. Our estimation of $e_{RS} = 12.6 \pm 1$ $\hbox {kCal}{\cdot }\hbox {mol}^{-1}$ gives a dissociation constant of $K_D = 1.3$ nM, which is in close agreement with experimental measurements ($K_D = 1.4-4.674$ nM). Furthermore, our analysis shows that only a reduced fraction (about 13) of the residues in the RBD interact during the protein adhesion. These residues play a critical role in the adhesion of the S-protein/ACE2 receptors, and can be used as a target for therapeutic strategies to prevent virus uptake in human cells. Finally, using a mechanistic model to analyze virus uptake, we concluded that S-proteins/ACE2 receptor’s binding has to be reduced at least by 50% to block the uptake of SARS-CoV-2 particles having radius between 30 to 70 nm. However, according to our analysis, the effectiveness of these strategies is strongly dependent on particle size and receptor density. Hence, such treatments might be more effective in blocking only a portion of the SARS-CoV-2 particles leaving others unaffected.

## Methodology

### Molecular dynamics simulations, Umbrella sampling and characterization

Molecular dynamics simulations were performed with the GROMACS software^16–18^. The molecular geometry was taken from different sources, including the protein data base models 6LZG for a single S-protein^19^, and the PDB file 6VYB full trimeric model^2^. In addition—since we started our simulations before the PDB models were available—we used the model provided by Smith et al.^7^ using the sequences available online (NCBI Reference: YP$\_$009724390.1) for the SARS-CoV S-protein’s crystal structure and the ACE2 receptor was generated using the PDB 2AJF file. While the actual numbers vary slightly, the trends are the same regardless the geometry. The model was loaded into GROMACS, where it was solvated in water using the TIP3P model to achieve a density of approximately $\rho =1000$ $\hbox {Kg}{\cdot }\hbox {m}^{-3}$. In order to allow for sufficient space for the pulling simulations, we generated computational cells with more than 1 nm between the proteins and end of the cells, and sufficient space on top to perform the pulling simulations. The biggest cell size has dimensions of $\sim 13.84 \times 14.99 \times 21.47$ (nm). After adding the solvent, the system had a non-zero charge and sodium (Na$^{+}$) ions were added as needed to equilibrate in all samples. All interatomic forces were computed with the CHARMM force-field^20^. The biggest cell had 648,265 atoms including proteins and solvent.

The solvated system was initially subjected to an energy minimization using a non-linear conjugate gradient. The forces were minimized with a convergence criterion of 1000 $\hbox {kJ}{\cdot }\hbox {mol}^{-1}{\cdot }\hbox {nm}^{-1}$. After the system was relaxed, it was subjected to an NPT ensemble for 1 ns using an initial temperature of $T=310.15$ K imposed with a Berendsen thermostat^21^. Pressure was controlled to 1 bar using a Berendsen barostat. The timestep was set to $\Delta t = 2$ fs. For all simulations carried in this work, short-range interactions were treated with a smooth force-switch cutoff of $r =1.2$ nm, and long-range electrostatics were treated using the Particle-Mesh-Ewald (PME) formalism, implemented in GROMACS^22^. Hydrogen-bonds were restrained with the LINCS algorithm^23^.

In order to compute the binding affinity between the S-proteins/ACE2 receptor, we used a combination of pulling simulations with umbrella sampling. The initial configuration of the S-proteins/ACE2 was subjected to a pulling simulation to generate the necessary configurations to perform an umbrella sampling. The pulling simulation was performed with an optimized spring constant of $K = 1300\hbox { kJ}{\cdot }\hbox {mol}^{-1}{\cdot }\hbox {nm}^{-2}$. This optimized spring constant was obtained by performing multiple umbrella samplings on the single S-protein-ACE2 receptor with spring constants in the range of $K =750-2000 \,\hbox {kJ}{\cdot }\hbox {mol}^{-1}{\cdot }\hbox {nm}^{-2}$, and optimizing the value using a quadratic fitting. Values obtained for different spring constants are shown in the **SI**. The pulling rate was set to $v_z = 5\hbox { nm}{\cdot }\hbox { ns}^{-1}$ along the z-direction and sampling simulations were run for a total time of $t = 10$ ns. The pulling direction was set such that the S-protein and ACE2 receptor were pulled apart from each other. The configurations generated along the pulling simulations were systematically used to generate trajectories for the umbrella sampling, described below.

Umbrella simulations were performed for configurations separated about $\Delta \lambda = 0.1$ nm from the reference configuration. Each configuration was constrained with a spring constant of $K = 1300\hbox { kJ}{\cdot }\hbox {mol}^{-1}{\cdot }\hbox {nm}^{-2}$ and run for $t = 10$ ns. These simulations provided enough sampling to obtain the potential of mean force along the reaction path to display the evolution of the free energy of the system. The PMF was then estimated using the Weighted Histogram Analysis Method (WHAM)^24^ using a bootstrap analysis to estimate the uncertainty in the PMF. We used 100 different measures, using 200 binning spaces along the reaction coordinate $\lambda$. We performed several simulations with the backbone of the proteins fixed and without any fix conditions. We found that simulations without fixing the backbone produce excessive elasticity in the proteins and lead to higher free energies.

Analysis of the atomistic configurations was performed with GROMACS cluster analysis tool. We scanned the configurations with a root mean squared displacement between the range of $0.15-0.25$ nm^25^. The cluster analysis yielded between three to six cluster for the analyzed configurations. In all cases shown, the most populated cluster was used when analyzing the configurations. The configurations were then analyzed with the software Pymol.

### Mechanistic model of endocytosis

Gao et al.^14^ developed a model considering a spherical particle being attached to an infinite membrane. The fusion of the particle with the membrane is driven due a release of the binding energy-computed above—when the S-protein and receptors are linked. It is assumed that the membrane has an equilibrium concentration of receptors, $\xi _R$, and when the particle attaches, this concentration changes with time, e.g., $\xi (s,t)$, *s* being the arc-length. In particular, when a particle is attached to the membrane, the density of receptors, $\xi (s,t)$, matches to the density of spike proteins, $\xi _S$, and far away tends to the equilibrium concentration. Considering the bending energy of a lipid bilayer—characterized through its bending modulus *B* and curvature $\kappa = \frac{2}{R}-$, and the binding energy between S-proteins and receptors, ($e_{RS}$) one can write down the following free-energy for the endocytosis process as

1 $$\begin{aligned} F(t) = k_B T \bigg [ \int _0^{a(t)} \bigg ( \frac{1}{2} B \kappa ^2 - \xi _S e_{RS} + \xi _S \ln \bigg (\frac{\xi _S}{\xi _R}\bigg ) \bigg ) ds + \int _{a(t)}^{\infty } \xi \ln \frac{\xi }{\xi _R} \bigg ]. \end{aligned}$$

In Eq. (1), $k_B T$ is the thermodynamic factor with $k_B$ and *T* denoting the Boltzmann’s constant and the absolute temperature, respectively. By requiring that the rate of free energy reduction gained in the wrapping process exactly balance the rate of energy dissipation consuming during the transport, Gao et al. found that there exists an optimal wrapping radius of the particles and a minimum radius below the particle cannot be wrapped. The wrapping time can be found as

2 $$\begin{aligned} t_w = \bigg ( \frac{R}{\alpha \sqrt{D}} \bigg )^2, \end{aligned}$$

where *R* is the radius of the particle, $\alpha$ is the speed factor ($\alpha > 0$), and *D* is the diffusivity of the receptors in the membrane. The speed factor is found by solving the rate equation

3 $$\begin{aligned} e_{RS} - \frac{1}{2} \frac{B \kappa ^2}{\xi _S} - f(\alpha ) + \ln f(\alpha ) + 1 = 0, \end{aligned}$$

with

4 $$\begin{aligned} f(\alpha )= {\tilde{\xi }} + \frac{\alpha ^2 (1-{\tilde{\xi }}) E_1(\alpha ^2)}{\alpha ^2 E_1(\alpha ^2) - \exp (-\alpha ^2)}. \end{aligned}$$

The parameter ${\tilde{\xi }} = \frac{\xi _R}{\xi _S}$ defines the ratio between equilibrium receptor density in the membrane and the S-porteins in the virus particle. In the above expression, $E_1$ is the exponent integral function defined as

5 $$\begin{aligned} E_1(x) = \int _x^{\infty } \frac{\exp (-u)}{u} du. \end{aligned}$$

The model predicts a minimum radius for spherical particle given by

6 $$\begin{aligned} R_{min} = \bigg ( \frac{ 2B }{ \xi _S \big [ e_{RS} - {\tilde{\xi }} + \log { {\tilde{\xi }} } + 1 \big ]} \bigg )^{1/2}, \end{aligned}$$

and an optimal radius that is determined numerically. Gao *et al.* determined the wrapping time as a function of the particle radius as well as the optimal particle radius numerically.

### Parameters determination

SARS-CoV-2 virus particles of sizes between $R = 30 - 70$ nm have been reported^1,15^. This range indicates a wide range of particles, with an average size of $R_{ave} = 50$ nm. Microscopic images indicate that around $17\pm 2$ spike proteins in the circumference of the virus. An elementary analysis indicates that the density of spike proteins in the novel SARS-CoV-2 virus must vary between $\xi _S = 2280 - 3660$ $\mu \hbox {m}^{-2}$ (about $\sim 90$ spike proteins in the surface) when the average radius is taken. The computed density values are in close agreement to other coronaviruses^26^. The bending stiffness of lipid bilayers ranges between $6.16 - 18.5$ $\hbox {kCal}{\cdot }\hbox {mol}^{-1}$ ($10 - 30 ~k_B T$)^27–29^. Here, following previous works, we adopt an average value of 12.3 $\hbox {kCal}{\cdot }\hbox {mol}^{-1}$ (20 $k_B T$)^14,30^.

The density of ACE2 receptors on the cell membrane, at thermodynamic equilibrium, is $\xi _R$ from which we compute the dimensionless ratio ${\tilde{\xi }} = \frac{\xi _R}{\xi _S}$. Given the limited data available on the density of receptors on human cells $\xi _R$ is difficult to estimate, in particular because receptor density varies across cell types and depends on the specific receptor. Moreover, no specific data is available (to the author’s knowledge) about the density of ACE2 in epithelial cells in human lungs, the target of SARS-CoV-2.

Chen et al.^31^ measured a density of 480–640 $\mu \hbox {m}^{-2}$ for receptor of various species, while Damioli et al.^32^ measured a density of $\sim 4.8$ $\mu \hbox {m}^{-2}$ for VEGFR2 receptors. Based on these measurements, we estimated ${\tilde{\xi }}$ to vary between $0.1-0.0001$ and adopt these values in our simulations. The receptor diffusivity was taken as $D=0.01$ $\mu \hbox {m}^{2}{\cdot }\hbox {s}^{-1}$ an average value for most cells^14,30,32^.

## Supplementary information

Supplementary file1

Supplementary file2

Supplementary file3
