# Supplementary material for: Quantifying the adhesive strength between the SARS-CoV-2 S-proteins and human receptor and its effect in therapeutics
Source: Sci Rep. 2020 Oct 16;10:17538. doi: 10.1038/s41598-020-74189-4 (PMC7568573; doi:10.1038/s41598-020-74189-4)
Supplement: Supplementary file 1 — Supplementary file1 [file 41598_2020_74189_MOESM1_ESM.pdf]

Quantifying the adhesive strength between the SARS-CoV-2 S-proteins and human receptor and its effect in therapeutics

Mauricio Ponga

mponga@mech.ubc.ca

Department of Mechanical Engineering, University of British Columbia, 2054 - 6250 Applied Science Lane, Vancouver, BC, Canada, V6T 1Z4

Supplementary Information

Calculation of the dissociation constant K_D:

The dissociation constant can be computed once the binding energy is computed. The relation is given by $e_{RS} = -k_B T \log(K_D)$. Using $T = 310.15$ K, and e_{rs}=12.6 kCal·mol^{-1} we obtain $K_D = 1.31 \times 10^{-9}$ M.

Residue interacting during the umbrella sampling.

When λ=0.4 nm, ten residues in the S-protein were in contact with the ACE2 receptor. These were clustered near the two anchor points shown in the manuscript. In Figure SI1 we show the residues in both molecules for λ=0.3 and 0.4 nm. The ACE2 receptor is always shown in green and the S1-protein is shown in cyan. The residues are shown with sticks with the back bone in yellow and magenta for the ACE2 and S-protein, respectively.

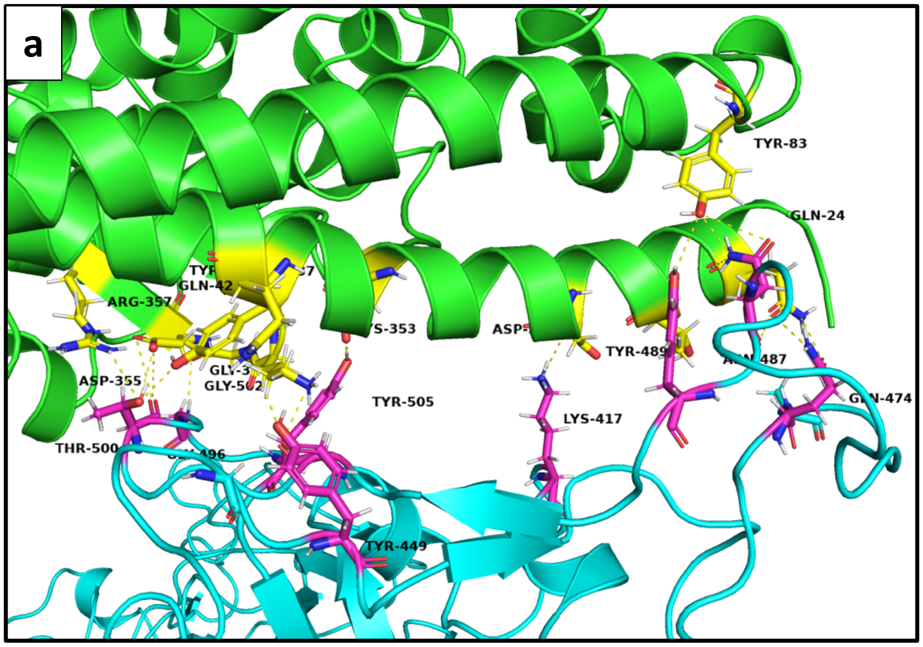

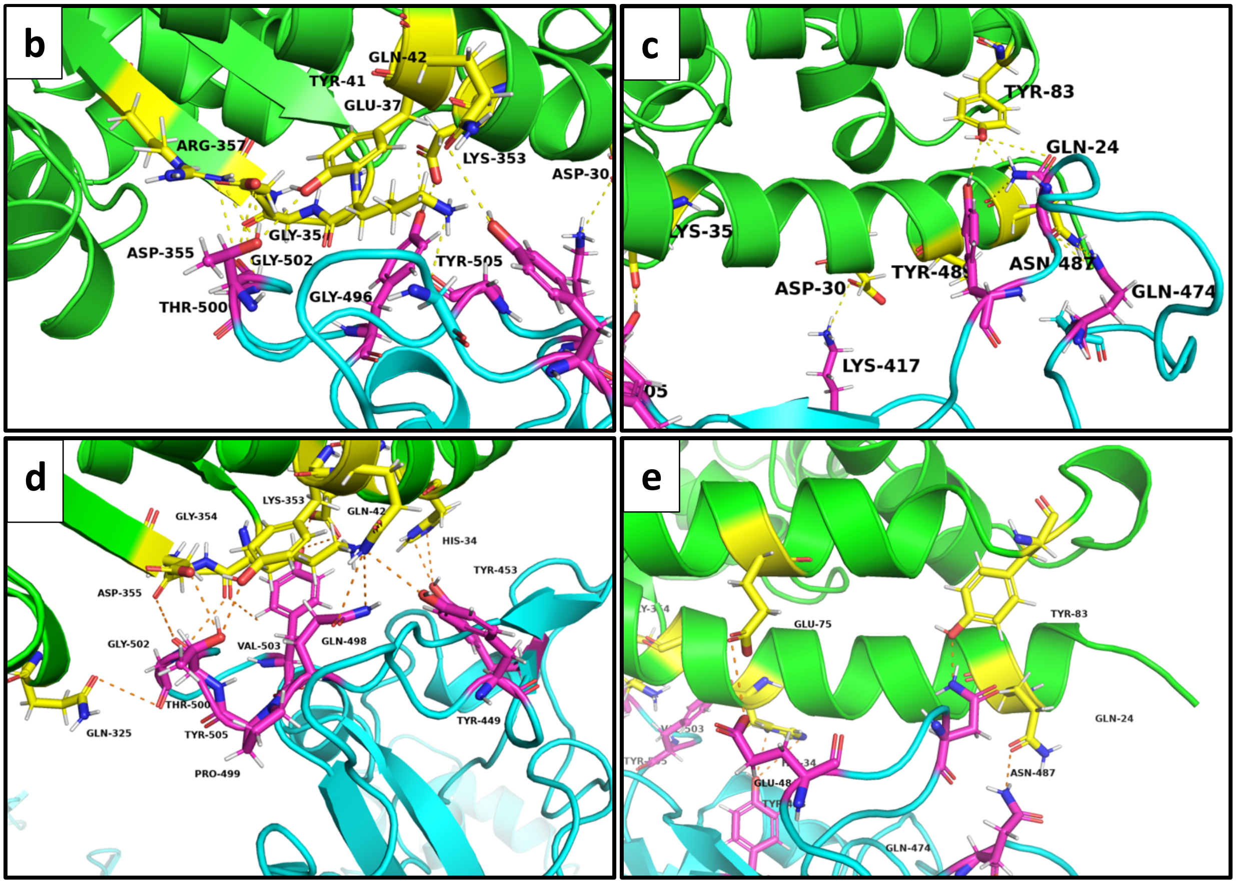

Figure SI1: Residues in contact during the bond-breaking simulation. (a) View of the RBD when λ=0.3 nm (b) Detail view of the left-hand side area in (a), and (c) view of the left-hand side area in (a). (d) and (e) shows the contact when λ=0.4 nm.

Table SI1: Residues active during the stretching between SARS-CoV-2 spike protein and ACE2 receptor.

λ [nm]	Residues in the S-protein	Residues in the ACE2 receptor
λ= 0.3	K-417, Y-449, Q-474, N-487, Y-489, G-496, T-500, G-502, Y-505	Q-24, D-30, E-37, Y-41, Q-42, Y-83, K-353, G-354, D-355, R-357
λ= 0.4	Y-449, Y-453, Q-474, E-484, N-487, Q-498, P-499, T-500, G-502, Y-505	Q-24, H-34, E-37, Y-41, E-75, Y-83, M-323, K-353, G-354, D-355, Q-325
λ= 0.8	Q-474, N-487, Q-498	Q-24, H-34, Y-83
λ= 1.4	E-484	Q-24
λ= 1.5	None	None

Wrapping time as a function of radius:

The model used to asses he effectivity of viral treatments allow us to quantify the wrapping time for particle uptake. Below, we provide the uptake time for four different dimensionless densities

$\tilde{\xi}$. As we can see from the graphical representation, the wrapping time is minimum for the highest receptor density.

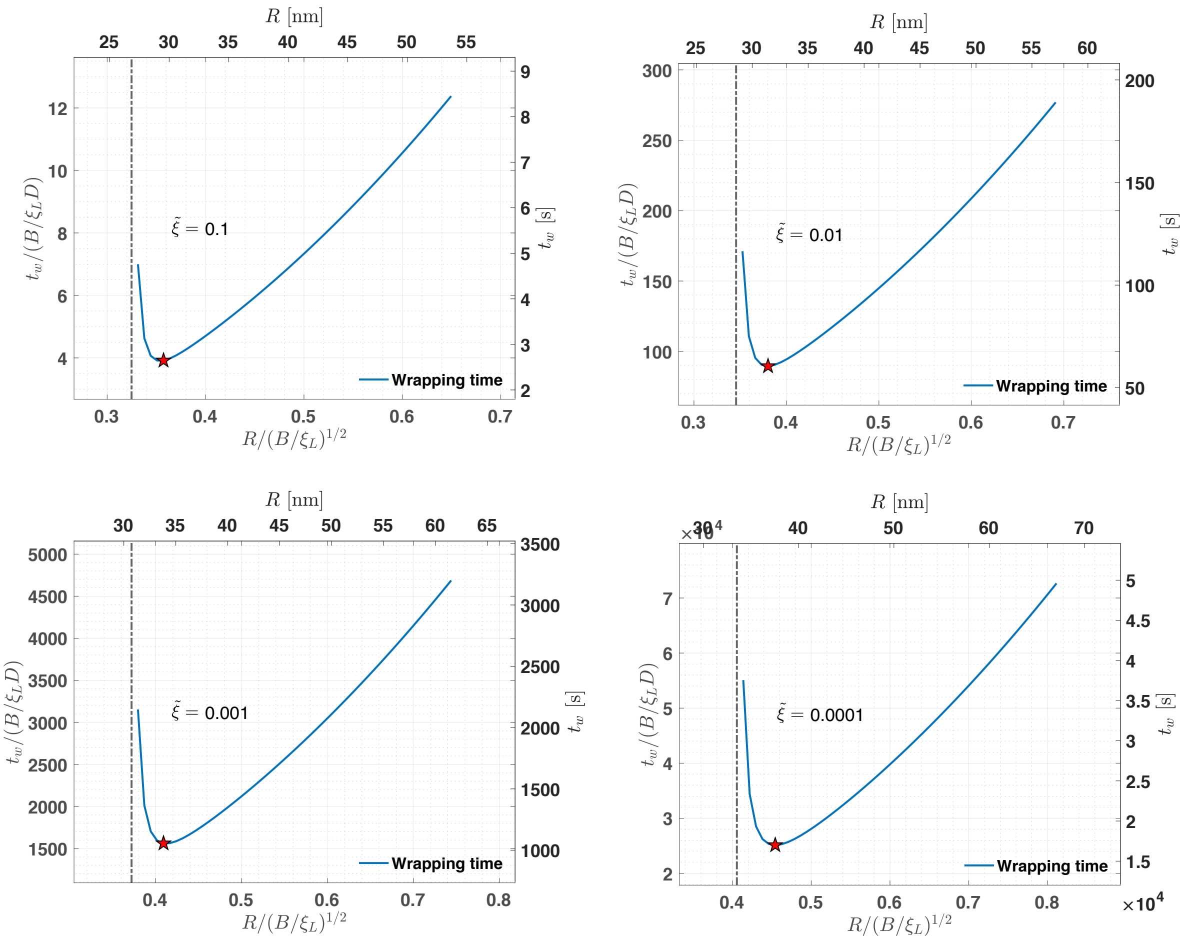

Figure SI2: Wrapping time for a particle of different radius using the parameter described in Table 1 and a dimensionless ligand density between $\tilde{\xi} = 0.1 - 0.0001$. The vertical asymptote illustrated with a dashed line indicates the minimum particle radius, R_{min}. The start indicates the *optimal radius* for which the wrapping time is minimum. The vertical dashed line indicates the minimum radius below a particle cannot penetrate the membrane. Optimal times vary between 2.75 s, 60 s, 1000 s, and 15,000 s depending on $\tilde{\xi}$.

_k_-factor effect in wrapping time:

It is also interesting to look at the change in the wrapping time with respect to reduced binding energies. This effect is shown in **Figure SI3** where the ratio $\frac{t_w^k}{t_m^{min}}$ is shown, where t_w^k is the wrapping time for a given *k*-factor, and t_m^{min} is the minimum wrapping time when *k*=1. We see again that a reduction of 30% in the binding energy (*k*=0.7) leads to an increase in the wrapping time between 1.5-2.0 times for receptor densities between $\tilde{\xi} = 0.1 - 0.001$. On the other hand, when the binding affinity is reduced by 50%, the wrapping time increases between 3-5 times with respect to the reference. This analysis indicates that reducing the binding affinity between the S-

proteins and ACE2 receptors impacts on both a reduction of the particle size that are allowed to penetrate the membrane and in the wrapping time.

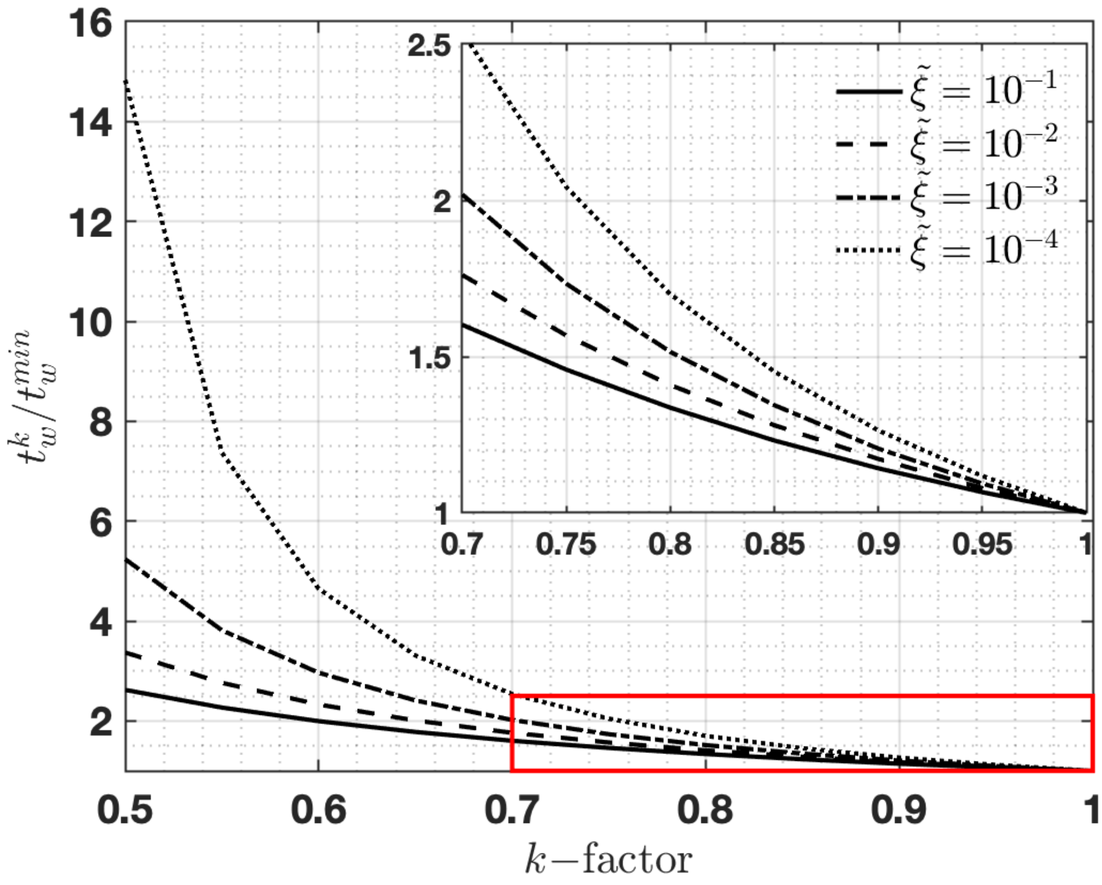

Figure SI3: Effect of reduced binding affinity (*k*-factor) in the minimum radius for wrapping. Four receptor densities were used, namely $\tilde{\xi} = 0.1$ (solid line), $\tilde{\xi} = 0.01$ (dashed line), $\tilde{\xi} = 0.001$ (dashed-dotted line), $\tilde{\xi} = 0.0001$ (dotted line). Wrapping time increases with reduced binding energy.

Equilibration of the sample, pulling simulation and umbrella sampling.

For completeness, we show the key relevant parameters and quantities in our simulations before performing the pulling and umbrella simulations. We next provide details on the energy convergence and thermalization of the sample. The energy minimization after solvation is shown Figure SI2.

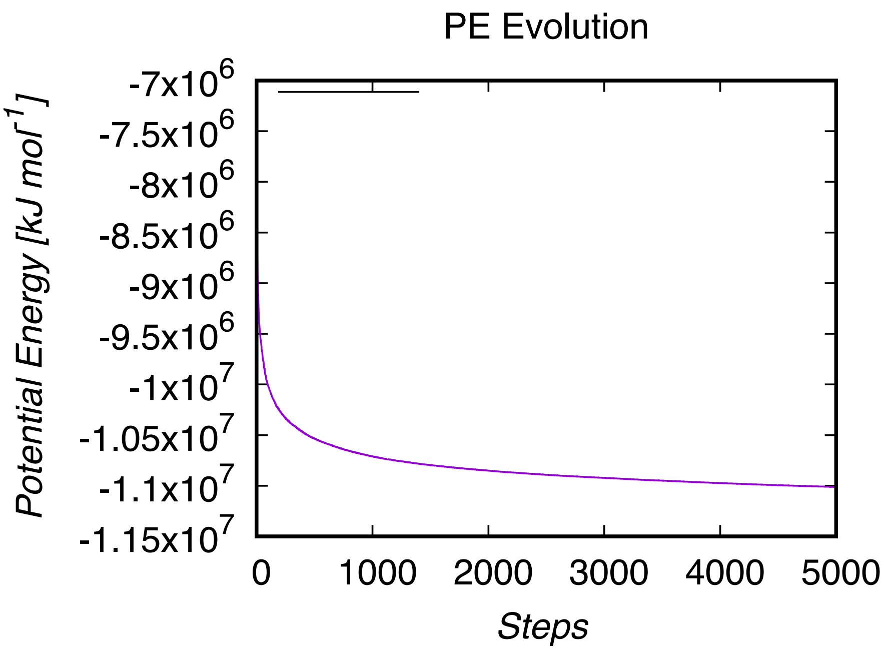

Figure SI4: Energy minimization for the full trimeric S-protein and ACE2 receptor solvated in water.

After the minimization procedure, an NPT ensemble was used to thermalize and stabilize the computational cell to T=310.15 K, and 1 bar. Below, the pressure and temperature evolution are shown during the thermalization process during 1000 ps (1 ns).

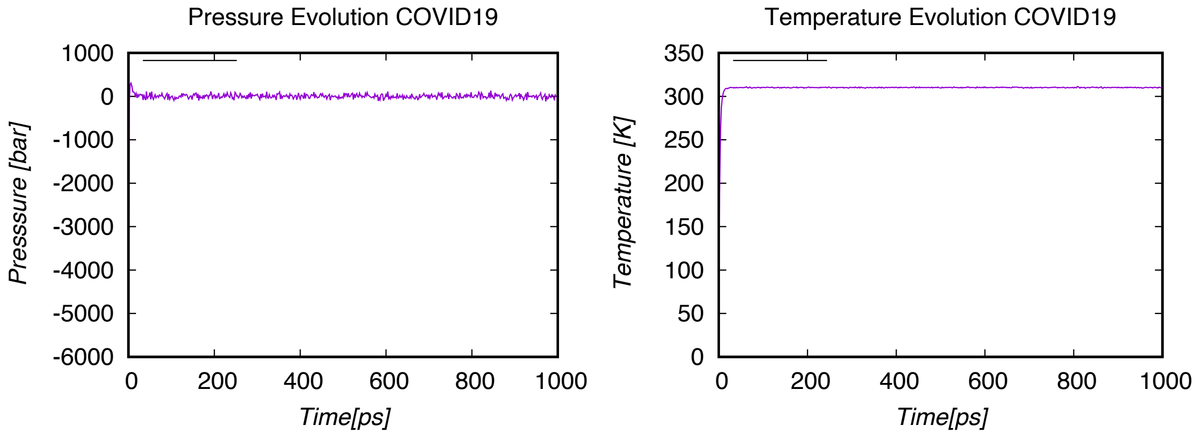

Figure SI5: Pressure and Temperature evolution during the thermalization of the sample.

Pulling simulations: After the thermal equilibration of the sample, we performed a pulling simulation using the center of gravity of these two molecules. The evolution of the distance between the center of gravity and pulling force are monitored below.

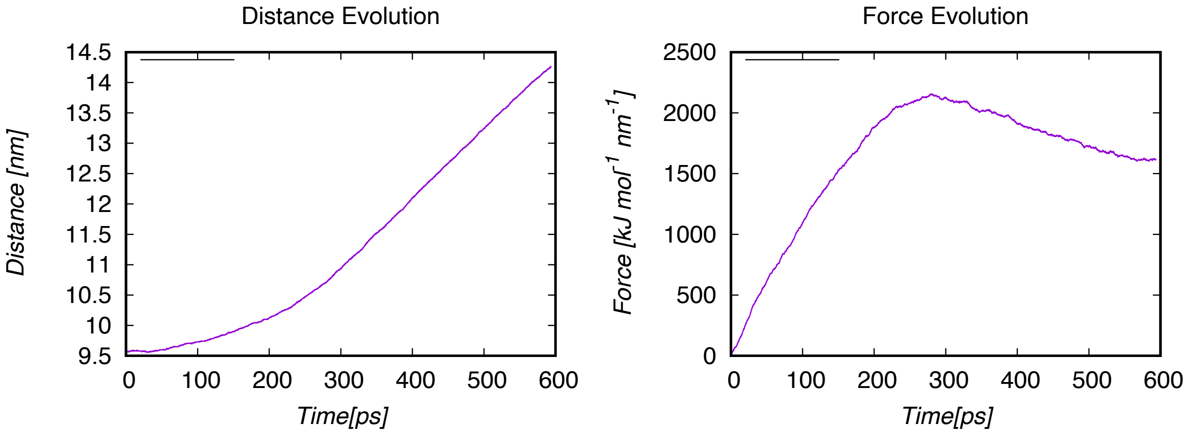

Figure SI6: Evolution of the distance and force as a function of time during the pulling simulation.

The preceding simulations generate a set of configurations along the *reaction path*. These configurations were extracted from the trajectory file, for their posterior analysis with GROMACS and umbrella sampling. We have tested different spring constant values, including 750, 1000, 1300 and 2000 *kJ* mol^{-1} nm^{-2}. The optimal value was obtained to be K = 1300 *kJ* mol^{-1} nm^{-2}, which was subsequently used in the full-scale model. **Figure SI7** shows the PMF for different values of the spring. Once we obtained the best spring value, we performed refined umbrella simulation, using a resolution of 0.1 nm or less across the reaction path.

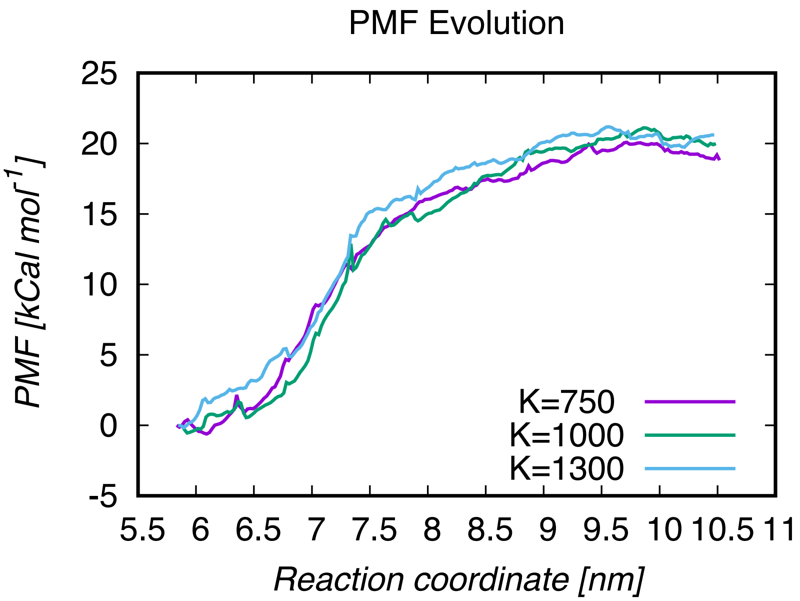

Figure SI7: Evolution of the PMF for different spring constants. We see that the maximum PMF is found when K = 1300 *kJ* mol^{-1} nm^{-2}. We used this value for the subsequent simulations.

The plot below shows the umbrella sampling generated for one case, where we see significant overlap between the different sampling simulations. This fine sampling (~0.1 nm) allowed us to

generate smooth profiles and reduce the error bars for the PMF. Such a fine refinement allows us to obtain smooth and continuous PMF plots.

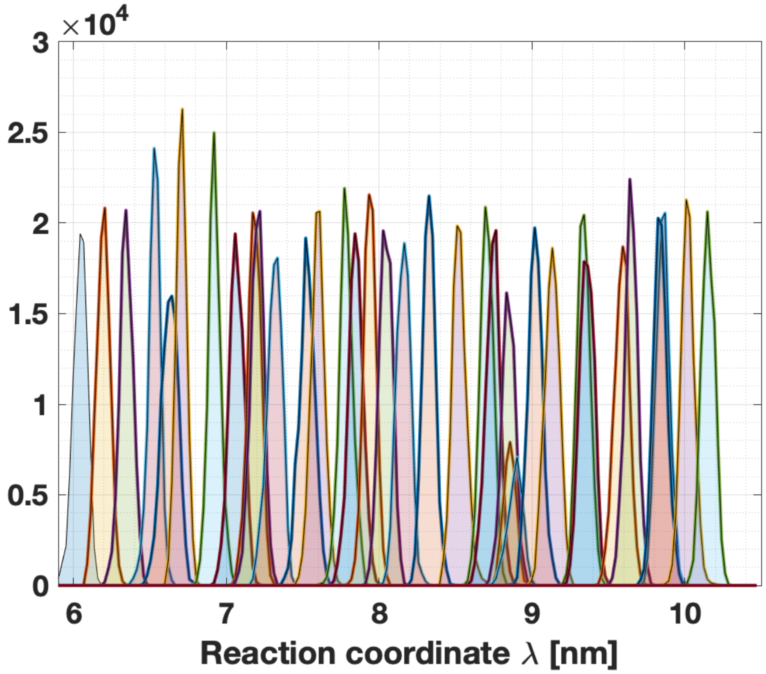

Figure SI8: Histogram obtained after performing multiple umbrella simulations. Some regions were poorly sampled and refined simulations were performed. The spatial resolution is 0.1 nm or better.
